# Intradialytic resistance training for short daily hemodialysis patients as part of the clinical routine: a quasi-experimental study

**DOI:** 10.3389/fragi.2023.1130909

**Published:** 2023-06-12

**Authors:** Victor M. Baião, Marvery P. Duarte, Vinícius A. Cunha, Gustavo Í. Dourado, Diogo V. Leal, João L. Viana, Antônio J. Inda-Filho, Otávio T. Nóbrega, Aparecido P. Ferreira, Heitor S. Ribeiro

**Affiliations:** ^1^ Faculty of Health Sciences, University of Brasília, Brasília, Brazil; ^2^ Research Center in Sports Sciences, Health Sciences, and Human Development, CIDESD, University of Maia, Maia, Portugal; ^3^ Interdisciplinary Research Department, University Center ICESP, Brasília, Brazil; ^4^ Post-graduation Program, Santa Úrsula University, Rio de Janeiro, Brazil

**Keywords:** exercise, dialysis, body composition, physical function, inflammation

## Abstract

**Background and purpose:** Hemodialysis patients have chronic systemic inflammation, musculoskeletal impairments, and body composition changes from several factors and exercise may attenuate. We evaluated the effects of an intradialytic resistance training program on body composition, physical function, and inflammatory markers in patients under short daily hemodialysis treatment.

**Materials and methods:** A quasi-experimental study in clinical routine was conducted over eight months. Measures of physical function (handgrip strength, five-time sit-to-stand, timed-up and go, and gait speed), body composition (by bioelectrical impedance), and inflammatory markers (interleukin [IL]-1 beta, IL-6, IL-8, IL-10, IL-12p70, and tumor necrosis factor-α) were assessed at baseline as well as at four and eight months past continued intervention. Patients underwent two intradialytic resistance training sessions per week supervised by exercise professionals.

**Results:** A total of 18 patients (62 ± 14 years; 55.6% ≥ 60 years; 44% female) were included. Significant increases in body mass index and basal metabolic rate were found at four and eight months compared to baseline. For physical function, timed-up and go performance improved at four and eight months compared to baseline. The other body composition and physical function measures, as well as all inflammatory markers, did not significantly change over time.

**Conclusion:** A supervised intradialytic resistance training program for patients on short daily hemodialysis treatment, as part of the clinical routine, may induce modest changes in body mass index, basal metabolic rate, and timed-up and go performance.

## 1 Introduction

Physiological changes in the metabolism of chronic kidney disease (CKD) patients are common (Stenvinkel and Larsson, 2013). In patients undergoing hemodialysis (HD), an increase in body fat and loss of muscle mass are prevalent conditions (Marcelli et al, 2016) leading to loss of functional independence, autonomy, and intolerance to daily living activities (Roshanravan, Gamboa and Wilund, 2017). Exercise intolerance deteriorates according to CKD progression and is associated with decreased kidney function, worsened quality of life, and increased mortality risk (Kirkman et al, 2021).

Chronic systemic inflammation is a common condition and related with impairment of protein biosynthesis in the muscle tissue (Kemp et al, 2004) as well as enhanced catabolism of nutritionally-related serum proteins (*e.g.*, albumin, transferrin, and pre-albumin), leading to loss of muscle mass (Ikizler et al, 2013; Sabatino et al, 2021), which changes the body composition and physical function. Thus, it is necessary to promote the stimulation of anabolic pathways to minimize the catabolic profile of CKD, providing maintenance of muscle skeletal and metabolic systems.

Regular exercise may play a role in decreasing and/or maintaining circulating levels of inflammatory markers, (Akchurin and Kaskel, 2015). Hence, intradialytic exercise is indicated as a non-pharmacological therapy for HD patients (Bennett et al, 2021), acting as a potential modifying of exercise intolerance and the inflammatory *millieu* (Liao et al, 2016; Bündchen et al, 2021). Despite different intradialytic exercise protocols have been extensively exploring general health benefits (Ferreira et al, 2021; Bernier-Jean et al, 2022), major these results were obtained in conventional HD treatment regimen without periodization and a few studies have explored intradialytic resistance training programs performed on the course of a short daily HD regimen.

This knowledge gap is important since exercise routinely prescribed seems to be a pivotal factor in this population. Therefore, we aimed to evaluate the effects of a supervised intradialytic resistance training program, as part of the clinical routine, on body composition, physical function, and inflammatory markers in patients under short daily HD treatment.

## 2 Materials and methods

### 2.1 Study design and participants

This is a longitudinal and quasi-experimental study in clinical routine that explored the effects of an 8-month supervised intradialytic resistance training program from a single dialysis center in Brasília, Brazil. Data was gathered between July 2019 and March 2020, prior to the COVID-19 pandemic. The study was approved by ethics committee in research of University Center ICESP (#2.497.191).

All participants enrolled in the intradialytic resistance training program signed the free and inform consent form and met the eligibility criteria of being or having: 1) adult or older adult (≥18 years); 2) under HD for over one month; 3) history of intradialytic hemodynamic stability; and 4) medical consent for the exercise program. Patients that changed dialysis regimen, underwent clinical surgery, had prolonged hospitalizations, and underwent lower limb amputation were excluded.

### 2.2 Hemodialysis treatment

Patients underwent HD therapy (Fresenius 5008, Oberursel, Germany) five to six times/week, with each dialysis session lasting for 2 h or 2 h30 min. The common access utilized was autogenous arteriovenous fistula, and the type of dialysis was conventional HD, not hemodiafiltration. Heparin was administered to the patients at the dosage of 100 IU/kg if not medically contraindicated, when heparin was substituted by oral anticoagulants. The dialysate temperature ranged from 35.5°C to 37°C during the sessions, without reutilization and filter FX 100 were used (Fresenius, Oberursel, Germany).

### 2.3 General procedures

Body composition, physical function, and inflammatory markers were examined at baseline, and after four and eight months on midweek dialysis sessions. Due to the clinical routine, the assessments of body composition and physical function were performed after dialysis sessions, conducted by the same exercise professional. Patients performed the resistance training sessions twice a week, with a total duration from 25 to 35 min per session, generally during the second HD hour, always supervised by an exercise professional (*i.e.*, exercise physiologist and/or physiotherapist).

### 2.4 Intradialytic resistance training program

A detailed description of the intradialytic resistance training program may be consulted elsewhere (Ribeiro et al., 2022c). In brief, patients underwent two resistance training sessions per week. All exercise sessions were supervised by an exercise physiologist or a physiotherapist. The training sessions consisted of a warm-up, including three to 5 minutes of joint mobility exercises, the conditioning part, with three lower-limb and three upper-limb resistance exercises (see Supplementary Figure SA1 for the videos), and three to five minutes of cool-down, consisting of two breathing exercises. The resistance exercise protocol consisted of one to three sets with 11–15 repetitions with a 1-min rest interval between sets and exercises (detailed in Supplementary Table SA1). Ankle weights and dumbbells were used. The six exercises were modified when the patients reached four months of intervention to avoid a training *plateau* and lack of motivation, as well as adjustments in range of motion, angle of the patient’s chair, and load were used to enable an individualized protocol. The exercise training protocol flowchart is shown in Supplementary Figure SA2. Moreover, the total training load for each intradialytic resistance training session was calculated as the number of sets x number of repetitions x total weight (sum of dumbbell and ankle weights) (Borresen and Lambert, 2009). Finally, the exercise session was interrupted in case of intradialytic complications such as hypotension and hypoglycemia (for more information, check Ribeiro et al., 2022c).

### 2.5 Body composition

Body composition was evaluated using the body composition monitor (BCM - Fresenius Medical Care, Germany) previously calibrated according to the manufacturer’s specifications. Initially, patients were asked to remove all metallic objects from their shirts and pants. All results were recorded in the BCM software (Fresenius Medical Care, Fluid Management Tool v.3) for further analysis. Body mass index (BMI), muscle mass, fat mass, body fat, basal metabolic rate (BMR), and total body water values were estimated and recorded.

### 2.6 Physical function

Handgrip strength (HGS) was evaluated with a handheld, hydraulic dynamometer (Saehan Corporation, Yangdeok-Dong, Korea). It was assessed in the arm without fistula, or the dominant arm if catheter access. Patients were seated with the shoulder in a neutral position, elbows flexed at a 90° angle, and wrist in a neutral position. During the evaluation and familiarization, participants received verbal encouragement. A total of three measurements were recorded with a 1-min rest period, and the highest value was recorded (Ribeiro et al, 2020).

The five-time sit-to-stand test (STS-5) was applied at a 46-cm seat to evaluate the strength of leg muscles. After familiarization, participants were asked to rise five times from a seated position without using their arms as quickly as possible. Time to completion in seconds was recorded (James, Schwartz and Orkaby, 2021; Duarte et al, 2022).

The timed up and go (TUG) was applied to evaluate mobility and physical performance. Three meters was marked off on the floor in front of a stable chair with arms (seat height of 46-cm); a large cone was placed on the mark of 3 m from the chair. Patients were instructed to stand up, walk as quickly and comfortably as possible to the cone on the floor, walk around the cone, come back, and sit back in their chairs. A familiarization trial was performed and then followed by a recorded trial (Wall et al, 2000).

Usual gait speed was measured in patients walking for 4 m. The test was performed twice, and the distance of 4 m was marked off on the floor to demarcate the route to be covered by the patient. The time it took to cover the route at the usual speed was recorded and the shortest time between two measurements was considered. The result was expressed in meters per second (m/s) (Mehmet, Robinson and Yang, 2020; Duarte et al, 2022).

### 2.7 Inflammatory markers

Blood was drawn through an arteriovenous fistula or venous catheter before the beginning of the first dialysis session of the week at baseline, four, and 8 months. Patients did not exercise in the 24 h preceding collections. Blood was stored into 4 mL K3EDTA tubes (13 × 100 mm) and centrifuged to separate the serum. After separation, serum was pipetted, stored, and identified into a 1.5 mL microtube (Eppendorf^®^) and subsequently frozen at −80°C in a vertical ultra-freezer (CL 120–80, ColdLab^®^) before further analysis.

The C-reactive protein (CRP) concentration was evaluated through routine clinical laboratory tests, following the same previous procedures but conducted at an associated laboratory of the dialysis clinic. Interleukin (IL)-1 beta (β), IL-6, IL-8, IL-10, IL-12p70, and tumor necrosis factor-α (TNFα) concentrations were assessed by a multiplexed flow cytometry method using one set of bead-based immunoassay known as the Human Inflammatory kit manufactured by the BD Biosciences^®^ (San Diego, CA, United States of America). Briefly, the lyophilized cytokine standards and the serum samples were processed, and the results were acquired using the BD FACSCalibur flow cytometer, FL4 channel. Three hundred events were acquired for each cytokine bead used. Data were analyzed using the FCAP software, version 3.0 (BD Biosciences^®^, San Diego, CA, United States of America). Standard curves for each cytokine were generated using a standard mixture of mediators supplied. The concentration in each serum was determined by interpolation from the corresponding standard curve. All collections and assessments were conducted by trained professionals with previous experience.

### 2.8 Statistical analyses

To describe the characteristics of the sample, descriptive statistics with frequencies, mean and standard deviation were used when there was normality. Non-parametric variables were expressed as the median and interquartile range (IQR). Data distribution was verified by the Shapiro-Wilk test. Depending on the data distribution, the Wilcoxon or paired *t*-test was used to assess differences between two moments.

The one-way analysis of variance (ANOVA) or Friedmann test for repeated measures were used to assess differences in the three moments. When there was a significant within-group effect (*p*-value < 0.05), the Bonferroni *post hoc* comparison test or the Friedmann two-way ANOVA by ranks were used to indicate group differences. The intention-to-treat analysis was used for patients who did not complete the whole intervention. Data imputation by the last observation carried forward method was used for missing data. All analyses were performed using the Statistical Package for the Social Sciences (version 26.0, SPSS Inc., Chicago, United States of America) and GraphPad Prism (version 8, GraphPad Software, San Diego, United States of America). Two-tailed tests were applied and a *p-*value < 0.05 was considered statistically significant.

## 3 Results

### 3.1 Baseline characteristics

**FIGURE 1:**
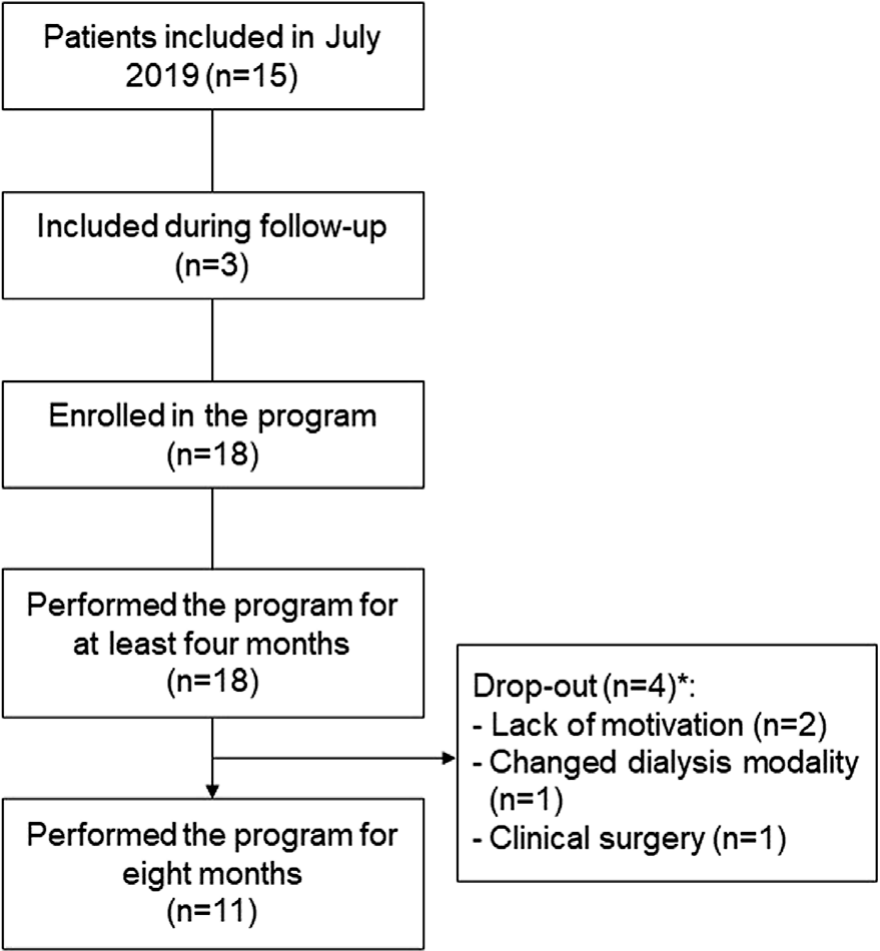
Flowchart of the clinical routine study. * intention-to-treat analysis was applied.

A total of 18 patients were followed during this clinical routine study of which 10 (55.6%) were elderly. Fifteen were included and started in July 2019 and three were included during the follow-up (Figure 1). All patients completed 4 months of regular exercise, while 11 participated in the exercise intervention for 8 months. Baseline characteristics are presented in Table 1. Diabetes mellitus was the main CKD etiology (n = 9, 50%), and the most prevalent comorbidity was hypertension (n = 11, 61%).

**TABLE 1:** Characterization of the short daily hemodialysis patients (n = 18).

Variables	
**Clinical, mean ± SD**	
Age (years)	62.1 **±** 13.9
Weight (kg)	67.6 **±** 10.1
Height (m)	1.63 **±** 9.2
Systolic blood pressure (mmHg)	134.3 **±** 29.9
Diastolic blood pressure (mmHg)	65.3 **±** 12.6
Resting heart rate (bpm)	76.2 **±** 10.5
Dialysis vintage (months), median [IQR]	19.0 [3.0–65.5]
**Gender, n (%)**	
Male	10 (55.6)
Female	8 (44.4)
**CKD etiology, n (%)**	
Diabetes mellitus	9 (50.0)
Glomerulonephritis	3 (16.7)
Hypertension	1 (5.6)
Polycystic kidneys	1 (5.6)
Unknown	4 (22.2)
**Comorbidities, n (%)**	
Hypertension	11 (61.1)
Diabetes mellitus	9 (50.0)
Cardiovascular disease	3 (16.7)
Asthma	1 (5.6)
Others	2 (11.1)

CKD: chronic kidney disease; SD: standard deviation; IQR: interquartile range.

### 3.2 Effects of the intradialytic resistance training program

Table 2 shows the comparison of body composition, physical function, and inflammatory markers over the intervention. Significant increases in BMI and BMR were found at four and eight months compared to baseline. For physical function, TUG performance improved at four an 8 months compared to baseline. The other body composition and physical function, as well as all inflammatory markers did not significantly change over time.

**TABLE 2:** Comparison of the body composition, physical function, and inflammatory markers over the intradialytic resistance training program.

Variables	Baseline	4-month	8-month	*p*-value
**Body composition**				
Weight (kg)	67.6 ± 10.1	69.7 ± 8.5	70.3 ± 9.2	0.146
Body mass index (kg/m^2^)	25.3 [23.4–26.6]	26.1 [24.9–27.7]^a^	25.9 [24.1–28.1]^a^	**0.030**
Lean mass (kg)	44.2 ± 6.9	45.7 ± 6.0	46.2 ± 6.9	0.131
Fat mass (kg)	22.8 ± 6.0	25.0 ± 6.3	25.0 ± 6.3	0.220
Body fat (%)	33.2 ± 6.2	33.4 ± 5.7	33.6 ± 6.2	0.822
Basal metabolic rate (kcal)	1363 [1255–1399]	1410 [1302–1458]^a^	1418 [1274–1527]^a^	**0.004**
Total water (L)	31.0 [28.7–32.9]	32.3 [30.3–35.4]	32.5 [29.8–36.7]	0.065
**Physical function**				
Handgrip (kgf)	24.8 ± 7.3	27.2 ± 9.0	27.3 ± 9.7	0.084
Five-time sit-to-stand (s)	16.2 ± 6.1	15.5 ± 4.9	15.1 ± 4.9	0.661
Timed up and go (s)	12.0 [8.0–18.9]	11.6 [8.2–14.3]^a^	10.2 [8.1–13.3]^a^	**0.030**
Gait speed (m/s)	0.8 ± 0.4	1.0 ± 0.3	0.9 ± 0.3	0.053
**Inflammatory markers**				
C-reactive protein (mg/L)	3.3 [1.6–6.1]	2.7 [1.1–5.6]	2.9 [1.2–6.9]	0.920
IL-6 (pg/mL)^b^	43.0 ± 13.0	43.0 ± 13.0	47.4 ± 18.4	0.108
IL-8 (pg/mL)	64.9 [48.0–125.8]	67.4 [57.0–104.5]	70.4 [56.0–119.3]	0.529
IL-12p70 (pg/mL)	18.8 ± 1.8	18.8 ± 2.0	19.2 ± 2.2	0.679
TNF-α (pg/mL)	61.7 ± 3.5	62.2 ± 4.1	63.4 ± 3.7	0.224
IL-10 (pg/mL)	12.3 ± 1.7	12.4 ± 2.1	13.8 ± 2.8	0.072
IL-6/IL-10 (pg/mL)^b^	3.3 [2.8–4.0]	3.2 [2.7–4.6]	3.5 [2.7–4.8]	0.264
IL-1β (pg/mL)	27.1 ± 3.2	27.3 ± 4.9	27.0 ± 4.6	0.967

IL, interleukin; β, beta; TNF-α, tumor necrosis factor-alpha.

^a^ Indicates a statistically significant difference from baseline (*p*< 0.05).

^b^ Extreme outliers were removed from analysis.

Bold values mean a statistically significant *p*-value (<0.05).

**FIGURE 2:**
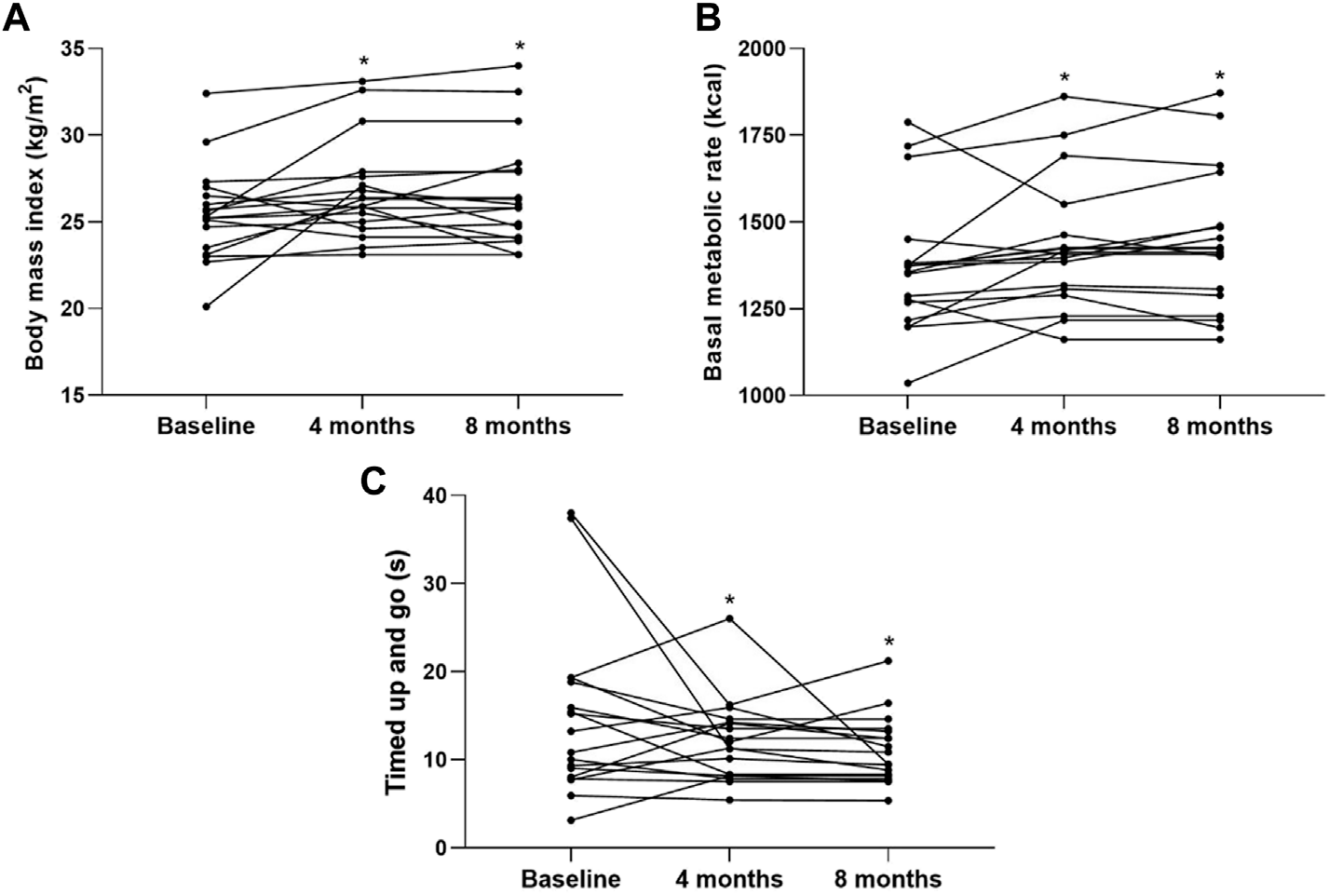
Individual changes from baseline to 4 and 8 months. **(A)** body mass index; **(B)** basal metabolic rate; **(C)** timed up and go.

**FIGURE 3:**
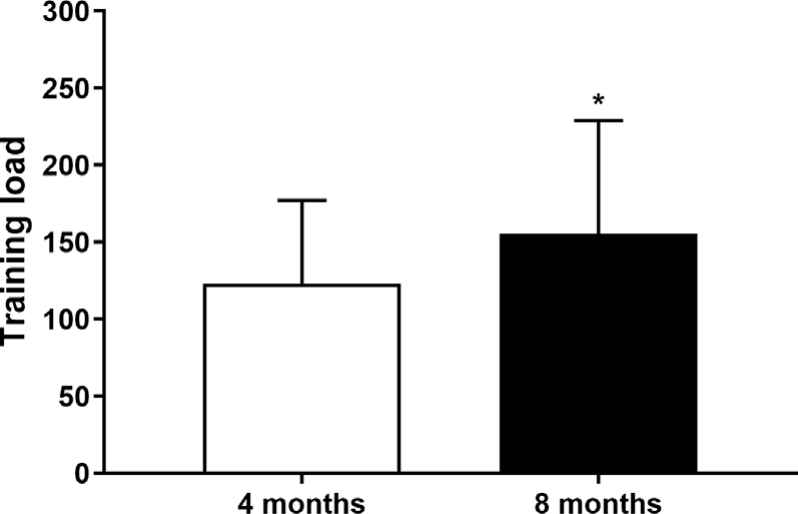
Training load over the intervention.

Figure 2 highlights the individual changes from baseline to four- and 8-month intervention periods for BMI, BMR, and TUG. In addition, Figure 3 shows that the training load significantly increased over the intervention (123.2 ± 54.1 versus 155.6 ± 73.4, *p* = 0.001).

## 4 Discussion

### 4.1 Main findings

The main findings of our study demonstrated that a supervised intradialytic resistance training program, as part of the clinical routine, promoted modest changes in BMI, BMR, and TUG performance of patients undergoing short daily HD. These findings highlight the importance of a structured intradialytic resistance program (Heitor S. Ribeiro, Andrade, et al, 2022b) in maintaining body composition, physical function, and inflammatory markers condition, which are particularly relevant in this population.

### 4.2 Body composition

The effects of intradialytic exercise on body composition are consistently observed in other works, showing clinical benefits to counteract muscle loss (March et al, 2022). Although muscle mass did not show a significant statistical increase in our study, this may not be considered a negative outcome in this population. Loss of muscle mass is a common feature and has been strongly associated with several clinical outcomes in HD patients (Ribeiro et al., 2022a). Considering the systemic catabolic condition, in which protein synthesis and protein degradation are affected, the maintenance of muscle mass (*i.e.*, non-reduction) in HD patients is clinically important. As expected, additional changes in BMI were experienced during intradialytic resistance training intervention. In this regard, such increase may not be interpreted as a negative outcome. Greater BMI has been consistently associated with survival in CKD patients, providing a protective effect (Kalantar-Zadeh et al, 2017). However, maintenance or gain in muscle mass can provide additional protective effects (Marcelli et al, 2015). Considering that HD patients frequently show an excess of body fat and intradialytic weight gain, and even if not elucidated, the changes arising from the HD modality (short daily) also seem to affect this variable (Kaysen et al, 2012; Zhu et al, 2019), these observations may not be a direct exercise-related effect.

### 4.3 Physical function

Another significant finding of our study was the improvement in the TUG test after four and 8 months. These findings are in agreement with previous studies which reported the positive effects of combined and resistance training on the improvement of functional mobility in dialysis patients (Frih et al, 2017; Hernández Sánchez et al, 2021). Noteworthy, low physical function in this population leads to clinical outcomes such as mortality (Ribeiro et al., 2022a). On the other hand, our continuous periodized and supervised intradialytic resistance training improved performance in TUG test, a marker of physical function. Thus, intradialytic resistance training may be a strategy to improve and/or mitigate the decline in physical function in HD patients.

### 4.4 Inflammatory markers

Our study revealed no significant changes in inflammatory markers after both four- and 8-month intervention periods. In the same way, Wilund et al (2010) did not see changes in any inflammatory markers after 4 months of intradialytic exercise training (Wilund et al, 2010). Other clinical trial with 12 weeks of intradialytic resistance training also reported no significant changes in such markers (Cheema et al, 2011). Indeed, inflammatory markers may be influenced by exercise protocol variations, length of exercise intervention, blood collection timepoint, and types of exercise, which show divergent results in the literature (Castaneda et al, 2004; Dong, Zhang and Yin, 2019; Moura et al, 2020; Bahat et al, 2021). Although we have not observed anti-inflammatory effects of intradialytic progressive resistance training in our sample, there was also no accentuation of the inflammaging process. Therefore, more robust studies with longer durations and stricter control of these variables are necessary to understand the effects of intradialytic exercise on pro- and anti-inflammatory mediators in HD patients.

### 4.5 Strengths and limitations

The present study has strengths to be highlighted. We showed that intradialytic resistance training is feasible to be implemented as a clinical routine in dialysis centers in a structured, systematized, and individualized approach. All the exercise sessions were conducted by exercise professionals (*i.e.*, exercise physiologists and physiotherapists). The expertise of exercise professionals is a determining factor in establishing a clinical exercise routine since they have specific skills and abilities for the prescription, progression, and monitoring of exercise programs (Wilund, Viana and Perez, 2020).

Nevertheless, there are limitations to be reported. We had a small sample size as the intervention was delivered in a single center. No dietary control was applied, but patients kept their dietary routines as recommended by the dietitians. Only one type of exercise with low to moderate volume, and no variation in weekly frequency was prescribed. Moreover, the absence of a control group may also be a limitation, however, having in mind that this is a clinical routine report, the objective of the study was to provide the exercise intervention as standard care for all patients. For this reason, three patients were included after the start of the study, which may also have influenced the results, but to minimize these effects, only the training period was considered, not the chronological study period. Thus, all data were analyzed by intention-to-treat, and the findings presented are possible outcomes arising from intradialytic exercise, but which need to be confirmed in more robust studies. Lastly, our HD regimen was short daily, and interpretation should not be extrapolated to other regimens.

## 5 Conclusion

In conclusion, we found that a supervised intradialytic resistance training program for patients on short daily HD treatment, as part of the clinical routine, may induce modest changes in BMI, BMR, and TUG performance. Further studies investigating the effects of exercise interventions with higher volume, intensity, weekly frequency, and variation in the type of exercises are necessary.

## Data Availability

The original contributions presented in the study are included in the article/Supplementary Materials, further inquiries can be directed to the corresponding author (HR).
