# Supplementary material for: Intradialytic resistance training for short daily hemodialysis patients as part of the clinical routine: a quasi-experimental study
Source: Front Aging. 2023 Jun 12;4:1130909. doi: 10.3389/fragi.2023.1130909 (PMC10291260; doi:10.3389/fragi.2023.1130909)
Supplement: Supplementary file 1 [file Table1.DOCX]

**Table A.1**. Intradialytic resistance training periodization during the 4- and 8-month intervention

| **Variables** | **1 to 4-week** | **5 to 18-week** | **19 to 27-week** | **28 to 34-week** |
| --- | --- | --- | --- | --- |
| **Intensity (RPE)** | 3 – 4 | 4 – 5 | 5 – 6 | 6 – 7 |
| **Sets (number)** | 1 – 2 | 2 | 2 – 3 | 3 |
| **Repetitions (number)** | 13 – 15 | 13 – 15 | 12 – 14 | 11 – 13 |
| **Rest interval (seconds)** | 60* | 60* | 60* | 60* |

RPE = rate of perceived exertion. *or until patient feel able and comfortable for a new set.
