# Supplementary material for: Intradialytic resistance training for short daily hemodialysis patients as part of the clinical routine: a quasi-experimental study
Source: Front Aging. 2023 Jun 12;4:1130909. doi: 10.3389/fragi.2023.1130909 (PMC10291260; doi:10.3389/fragi.2023.1130909)
Supplement: Supplementary file 2 [file DataSheet2.docx]

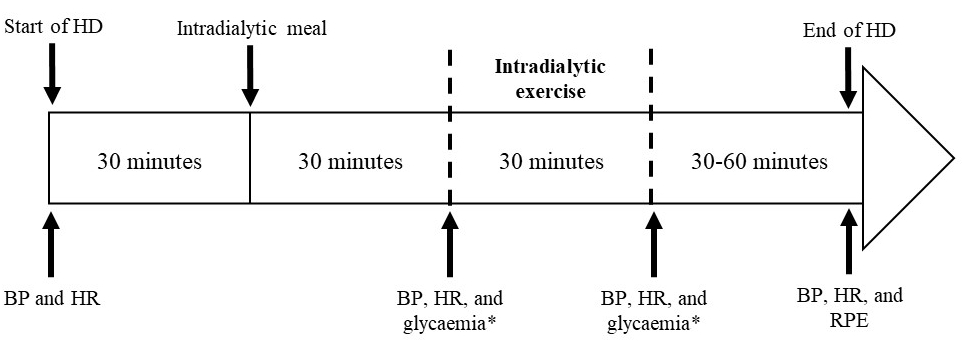


**Figure A.2**. Intradialytic resistance training protocol flowchart

BP = blood pressure; HD = hemodialysis; HR = heart rate; RPE = rate of perceived exertion. *only for the diabetic patients
