# Supplementary material for: Intradialytic resistance training for short daily hemodialysis patients as part of the clinical routine: a quasi-experimental study
Source: Front Aging. 2023 Jun 12;4:1130909. doi: 10.3389/fragi.2023.1130909 (PMC10291260; doi:10.3389/fragi.2023.1130909)
Supplement: Supplementary file 3 [file DataSheet1.docx]

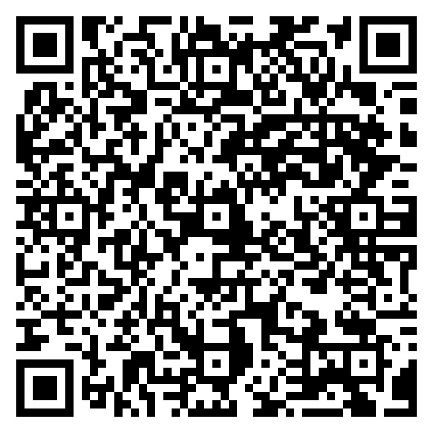


**Figure A.1.** QR code with some of the videos of the prescribed intradialytic exercises.

Link to access: <https://drive.google.com/drive/folders/1g9iIeMImmOYipROATekMRwPTcnhMSfTU?usp=sharing>
